# Self-assembling thermostable chimeras as new platform for arsenic biosensing

**DOI:** 10.1038/s41598-021-82648-9

**Published:** 2021-02-04

**Authors:** Rosanna Puopolo, Ilaria Sorrentino, Giovanni Gallo, Alessandra Piscitelli, Paola Giardina, Alan Le Goff, Gabriella Fiorentino

**Affiliations:** 1 Department of Biology, University of Naples Federico II, 80126 Naples, Italy; 2 Department of Molecular Chemistry, CNRS, University Grenoble Alpes, 38000 Grenoble, France; 3 Department of Chemical Sciences, University of Naples Federico II, 80126 Naples, Italy

**Keywords:** Molecular biology, Electrochemistry

## Abstract

The correct immobilization and orientation of enzymes on nanosurfaces is a crucial step either for the realization of biosensors, as well as to guarantee the efficacy of the developed biomaterials. In this work we produced two versions of a chimeric protein, namely ArsC-Vmh2 and Vmh2-ArsC, which combined the self-assembling properties of Vmh2, a hydrophobin from *Pleurotus ostreatus*, with that of *Tt*ArsC, a thermophilic arsenate reductase from *Thermus thermophilus*; both chimeras were heterologously expressed in *Escherichia coli* and purified from inclusion bodies. They were characterized for their enzymatic capability to reduce As(V) into As(III), as well as for their immobilization properties on polystyrene and gold in comparison to the native *Tt*ArsC. The chimeric proteins immobilized on polystyrene can be reused up to three times and stored for 15 days with 50% of activity loss. Immobilization on gold electrodes showed that both chimeras follow a classic Langmuir isotherm model towards As(III) recognition, with an association constant (K_AsIII_) between As(III) and the immobilized enzyme, equal to 650 (± 100) L mol^−1^ for ArsC-Vmh2 and to 1200 (± 300) L mol^−1^ for Vmh2-ArsC. The results demonstrate that gold-immobilized ArsC-Vmh2 and Vmh2-ArsC can be exploited as electrochemical biosensors to detect As(III).

## Introduction

Arsenic (As) is a toxic metalloid widespread in soil, water and air, harmful to humans and the environment^1,2^; due to its toxicity, one biotechnological challenge is centred on the development of biosensors to monitor its concentration in the environment^3^. Among species found in natural waters, the inorganic forms of As(III) and As(V) are those predominant with enhanced toxicity and greater mobility^4^. Microbial activities play important roles in the mobilization of arsenic^5–7^, and comprehensive knowledge on the molecular basis of arsenic metabolism/tolerance is a key step for developing efficient and selective arsenic biosensors.

The arsenic resistant thermophilic bacterium *Thermus thermophilus* HB27 owns an arsenate reductase (*Tt*ArsC) capable of reducing As(V) to As(III), which is then extruded outside the cell by a membrane P_1B_ type ATPase^8^; both proteins are finely regulated at transcriptional level by the metal responsive transcriptional repressor *Tt*SmtB^9,10^. *Tt*ArsC belongs to a subfamily of thioredoxin-coupled arsenate reductases, whose paramount protein is ArsC from *Staphylococcus aureus* plasmid pI258, characterised by the presence of three redox active cysteines, one performing the nucleophilic attack to the substrate and the other two that form a Cys–Cys disulphide bond upon reduction of As(V) into As(III)^11^. Members of this subfamily are also endowed with phosphatase activity because they contain a P-loop (CTHNSAR) homologous to that of low molecular weight protein tyrosine phosphatases (LMW PTPase) whose consensus sequence is CXGNXCR. Messens and co-workers proposed that these ArsCs evolved from LMW PTPase by a change of mechanism, that maintained the oxyanion substrate binding^12^. Consequently, to perform reductase activity they require the action of disulphide cascades and interaction with thioredoxin reductase (Tr), thioredoxin (Trx) and NADPH as redox partners, while to perform phosphatase activity they use mainly amino acids of the phosphate binding loop^11^. During the reduction reaction, a major conformational change occurs in ArsC upon oxidation, which is required to transport the oxidative equivalents from the As(V) in the P-loop to the surface of the enzyme^12^.

In *Tt*ArsC, the oxyanion binding site is conserved and includes the catalytic nucleophile Cys7, which is involved in both arsenate and phosphate binding. Nevertheless, its phosphatase activity is much weaker than that reported for ArsC from *S. aureus* pI258, probably because of differences in the amino acid composition of the active-site loop^13^. The other two conserved Cys residues (Cys82 and Cys89), essential for the reduction of the arsenate substrate, are spatially separated from the P-loop. To date, *Tt*ArsC is one of the most thermostable arsenate reductases characterised^13^.

Thermophilic enzymes/proteins are known to be more resistant to harsh conditions than their mesophilic counterparts, in fact they are often employed in many biotechnological applications^14–16^, such as degradation of polysaccharides of industrial interest^17,18^, antioxidants^19^, molecular biology tools^20,21^; and biosensing^8,22,23^. *Tt*ArsC, thanks to its thermophilic nature, is highly resistant to changes in pH, temperature and ionic strength, and was employed as component of an optical biosensor able to detect both As(V) and As(III) at low concentrations^24,25^.

In recent years it has emerged that a key step for increasing the performances of biosensors is represented by the biological interfacing of materials^26,27^. In particular, electrochemical biosensors are based on the efficient immobilization of enzymes on electrode surfaces. This immobilization technique must rely on effective functionalization strategies in order to preserve the recognition and/or catalytic ability of enzymes towards the targeted substrate^28–30^. In the case of enzyme-based arsenic biosensors, molybdenum-containing arsenite oxidases have been most exclusively investigated owing to its ability to electroenzymatically oxidize arsenite on electrodes^3,31–33^. Other examples of arsenic biosensors have been based on the inhibition properties of arsenic towards certain type of enzyme electrocatalytic activity^34^.

In bio-devices the specific properties and performances of bio/non-bio interfaces are also crucial features, therefore the development of biosensors based on suitably engineered self-assembling amyloid fibrils constitutes a promising opportunity to fulfil this task^35^. Self-assembling proteins are considered as a fundamental and green strategy to build hierarchical structures in hybrid functional assemblies^36^; in this context, hydrophobins (HFBs) are small fungal proteins (≈ 20 kDa) able to assemble spontaneously into amphiphilic monolayers at hydrophobic/hydrophilic interfaces. These amphiphilic proteins can be grouped into two classes based on the spacing of eight conserved cysteine residues and the nature of the amphipathic monolayers that they form. Class I HFBs are amongst the first proteins recognized as functional amyloids^37^; they form fibrillar structures which are extremely robust and can be disassembled only in strong acids. Vmh2 from *Pleurotus ostreatus*, is a class I HBF, known to self-assemble into stable films, able to change the wettability of surfaces and to strongly adsorb other proteins in their active form^38–41^. Thanks to its characteristics, Vmh2 has already been employed in the construction of chimeric proteins via genetic fusions with different proteins: the enzyme glutathione-*S* transferase (GST), which was used to quantify toxic compounds in aqueous environmental samples^42^; the green fluorescent protein (GFP) for the development of a biosensor to monitor thrombin in plasma samples^43^; the laccase POXA1b from *P. ostreatus* for the detection of phenolic compounds in different matrices^44^; and the antimicrobial peptide LL-37 for the development of anti-bacterial surfaces^45^.

In this context, this work aimed at realizing arsenic biosensing by developing a chimeric protein which combines the recognition properties and the straightforward stability of *Tt*ArsC with the self-assembling properties of the hydrophobin Vmh2.

## Results and discussion

### Design of chimeric proteins

In order to develop a biosensor with increased biosensing and surface adhesive properties, two different chimeric genes were designed: one coding for *Tt*ArsC at the N-terminal and Vmh2 at the C-terminal (ArsC-Vmh2), and a second coding for Vmh2 at the N-terminal and *Tt*ArsC at the C-terminal (Vmh2-ArsC). Both chimeric genes contained a sequence encoding a flexible linker of 15 amino acids between the two proteins, and a His-tag at the C-terminal, preceded by a thrombin cleavage site. The I-TASSER tool^46,47^ was used to generate the 3D models of chimeras, as well as those of the native Vmh2 and *Tt*ArsC. The confidence scores (C-score) of the predicted models for ArsC-Vmh2 and Vmh2-ArsC are − 3.72 and − 2.80, respectively, suggesting that they can be considered reliable.

**Figure 1:**
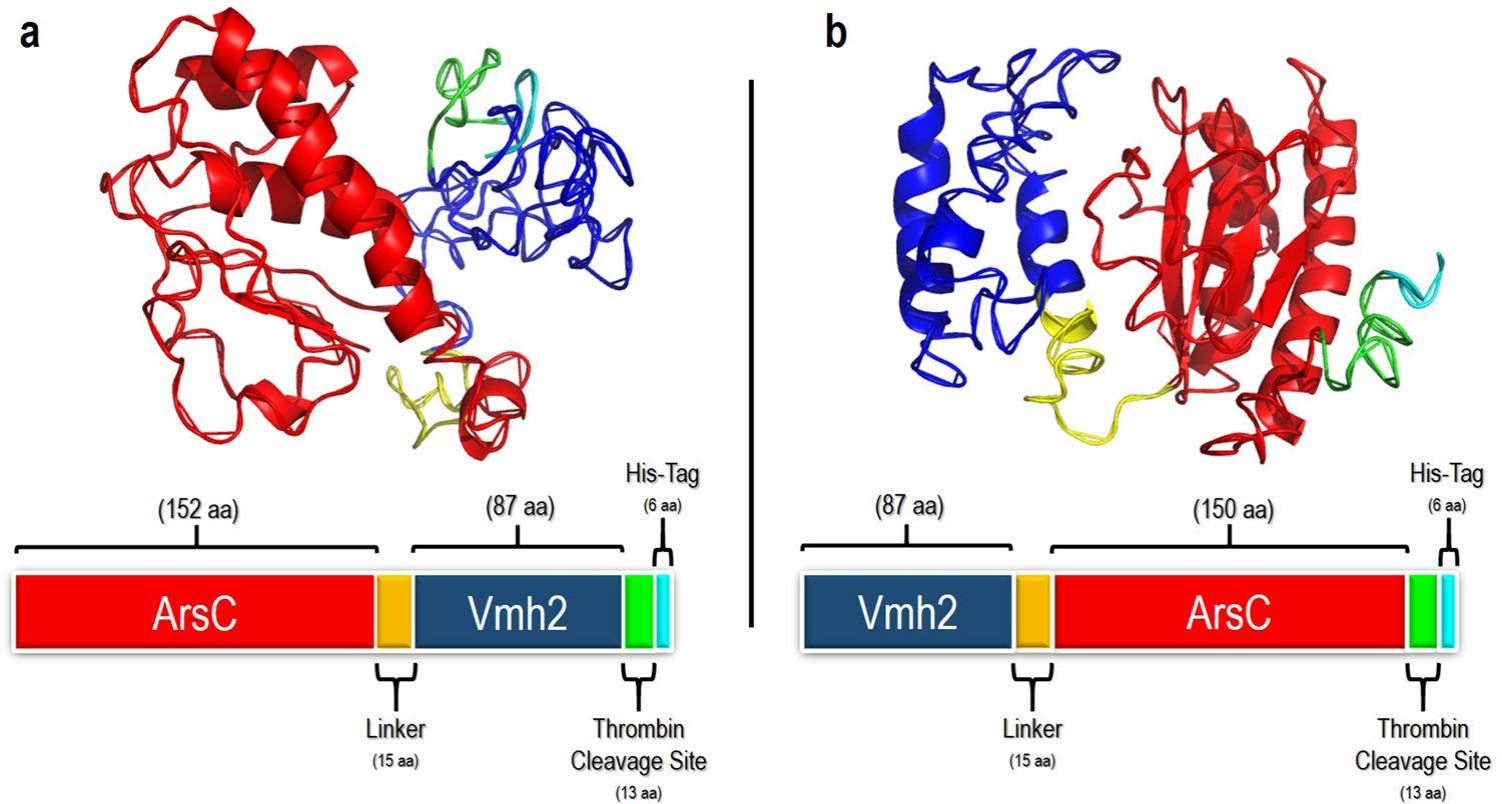
3D models and schematic organization of the chimeric proteins. (**a**) ArsC-Vmh2 and (**b**) Vmh2-ArsC. In both panels ArsC is reported in red; Vmh2 in blue; the linker in yellow, the thrombin cleavage site in green and the His-tag in ciano.

As shown (Supplementary Fig. S1a), the obtained model of HFB overlaps that of the native hydrophobin Vmh2, previously proposed by Pennacchio et al.^48^. The models of ArsC-Vmh2 and Vmh2-ArsC show that the arsenate reductase and the hydrophobin are independently folded in both the chimeras; the 3D model of *Tt*ArsC substantially overlaps the corresponding moiety in both chimeras (Supplementary Fig. S1b,c), suggesting that the folding of the arsenate reductase is preserved; on the other hand, ArsC-Vmh2 and Vmh2-ArsC models present differences in the Vmh2 region, which appears as a disordered loop in the first chimera, while it is organized in α-helixes in Vmh2-ArsC (Fig. 1). Moreover, Vmh2 folding in the models of both chimeras differ from that of the native HFB. Hydrophobins are known to be plastic and highly flexible proteins that can modify their structures upon changing external conditions or interaction with other proteins^49–51^; however, 3D structural studies are necessary to draw definitive conclusions on how Vmh2 folding influences the chimeras adhesive properties.

### Production of chimeric proteins

The recombinant protein production in *E. coli* was conducted inducing gene expression during the exponential growth phase. The estimated amount of protein present as inclusion bodies (mg/liter of culture) was ~ 24 mg for Vmh2-ArsC and ~ 12 mg for ArsC-Vmh2, respectively. To recover refolded proteins from the inclusion bodies, three different conditions were exploited (Table 3, “Methods” section): the best renaturation procedure resulted to be condition A, consisting in a refolding step in Tris–HCl (A_R_ and C_R_ refolding buffers), followed by dialysis in Tris–HCl (A_D_ dialysis buffer). Vmh2-ArsC is not only more expressed in *E. coli*, but it is also better recovered: a higher yield of refolded Vmh2-ArsC was obtained in comparison to ArsC-Vmh2 (~ 15.5 mg vs ~ 4.5 mg in condition A; ~ 4.1 mg vs ~ 1.1 mg in condition B; ~ 1.6 mg vs ~ 0.1 mg in condition C) (Supplementary Fig. S2a). The differences in expression and recovery yields can be explained by a diverse aggregation propensity of the unfolded chimeras as estimated by the TANGO tool^52–54^; in particular, ArsC-Vmh2 has a higher tendency to aggregation than Vmh2-ArsC; indeed, SDS PAGE analysis shows that the electrophoretic pattern of ArsC-Vmh2 contains bands at higher molecular weight in comparison to Vmh2-ArsC. Protein identity was also confirmed by Western blot analysis (Supplementary Fig. S2b,c and Supplementary Fig. S4). Since higher amounts of both refolded chimeras were obtained using a Tris–HCl buffer (~ 15.5 mg vs ~ 4.5 mg), all the following characterizations were performed on chimeras renatured using the condition A.

### Activity of the chimeric enzymes

**Figure 2:**
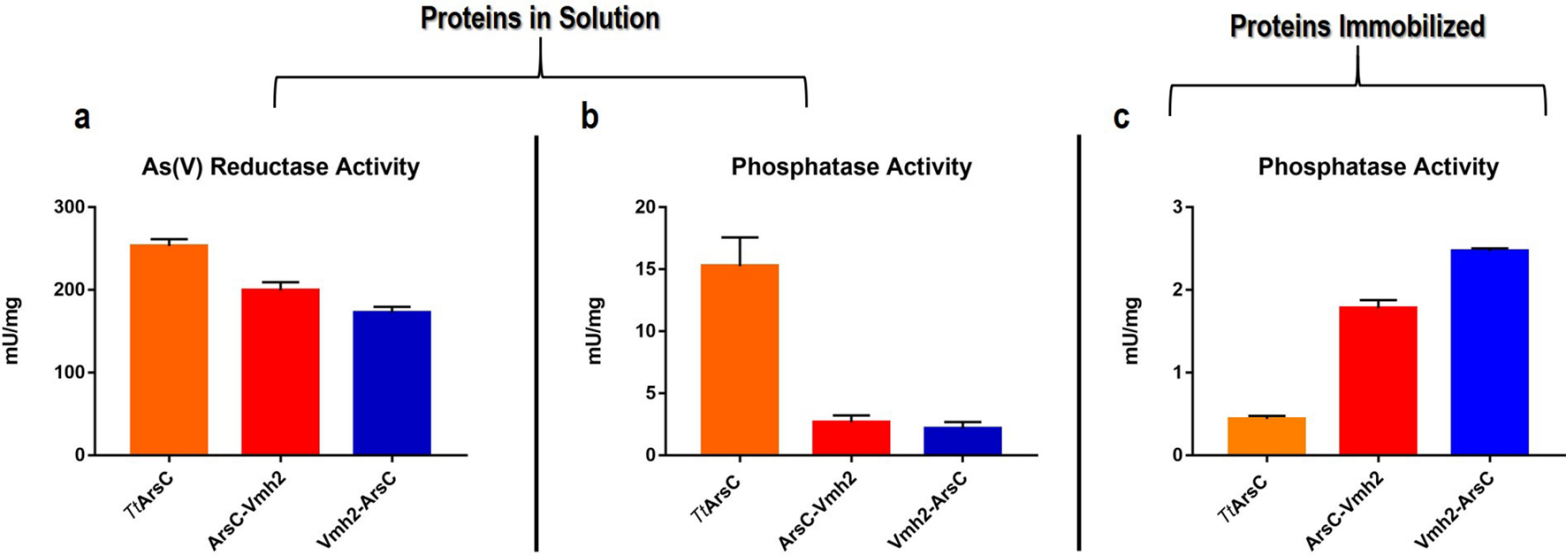
Graphical representation of the specific activities of *Tt*ArsC (yellow), Arsc-Vmh2 (red) and Vmh2-ArsC (blue). (**a**) As(V) reductase activity assay; (**b**) phosphatase activity assay; (**c**) phosphatase activity assay on the immobilized proteins.

In order to verify whether the chimeric proteins were active enzymes, arsenate reductase activity assays were performed using recombinant purified *Tt*ArsC as control^13^. Enzyme assay relies on the use of a redox cascade to recycle the enzyme. The enzymatic activity was measured by coupling the oxido/reducing system NADPH/Tr/Trx to chimeras and following the decrease of NADPH at 340 nm^12^. This assay was previously set up for *Tt*ArsC and use a hybrid system composed by the recombinant thermophilic *Ss*Tr of *S. solfataricus* and the recombinant Trx from *E. coli*, both stable at 60 °C^55,56^. Chimeric proteins exhibit a specific activity (ArsC-Vmh2 0.20 ± 0.01 U/mg; Vmh2-ArsC 0.17 ± 0.01 U/mg) comparable to that of *Tt*ArsC (0.25 ± 0.01 U/mg) (Fig. 2a; Supplementary Table S1).

The activity of the chimeras was also investigated through phosphatase activity assays using pNPP as substrate. The arsenate reductase moiety in the chimeras is in fact endowed with vestigial phosphohydrolase activity according to the hypothesis that the oxidoreductase function evolved from phosphatases in an oxidizing atmosphere^57,58^. This assay is much simpler and does not require other proteins. Notably, the phosphatase activity assay showed that both chimeras have similar specific activities (ArsC-Vmh2 2.7 ± 0.5 mU/mg; Vmh2-ArsC 2.2 ± 0.5 mU/mg) (Fig. 2b) but almost fivefold lower than that detected for *Tt*ArsC (15 ± 2 mU/mg) (Fig. 2b; Supplementary Table S1). This latter result highlights a difference in the activity of the chimeras in comparison to *Tt*ArsC, that was not observed when the activity was measured in the reductase assay. This fact can be explained hypothesizing that diverse conformational changes occur upon binding of the two substrates; in fact arsenate and the synthetic *p*-nitrophenylphosphate are structurally very different and can influence chimeras structural rearrangement in different way.

These results prove that the enzymatic properties of *Tt*ArsC are maintained in the chimeras and hence either ArsC-Vmh2 and Vmh2-ArsC are good candidates for arsenic sensing.

### Immobilization on polystyrene plate

In order to investigate on the adhesive properties of the chimeric proteins, we assessed their ability to adsorb on polystyrene; for this purpose, 100 μL of chimeras were spotted on a multiwell polystyrene plate at different concentrations and the amount of immobilized protein determined. The best immobilization conditions were chosen comparing the yields calculated as described below (Methods-Immobilization on polystyrene plate). Table 1 shows that both ArsC-Vmh2 and Vmh2-ArsC, despite their structural differences in the HFB domain, are adsorbed with 100% efficiency using different amount of proteins; as expected, this value is higher than that observed for native *Tt*ArsC (yield of immobilization 60% for 10 μg of protein) proving that Vmh2 moiety contributes to enzyme immobilization on hydrophobic surfaces.

**Table 1:** Yield of immobilization of ArsC-Vmh2 and Vmh2-ArsC compared to *Tt*ArsC on polystyrene plates.

Yield of immobilization (%)	(μg) spotted			
	2.5 (%)	5 (%)	10 (%)	20 (%)
ArsC-Vmh2	100	100	100	54 ± 5
Vmh2-ArsC	100	100	100	56 ± 5
*Tt*ArsC	100	76 ± 5****	60 ± 5****	N.A

*N.A.* not analysed. Statistical analysis was performed through the ordinary one-way ANOVA on GraphPad Prism 7.00; significant differences of TtArsC in comparison to chimeras are indicated as: *p < 0.05; **p < 0.01; ***p < 0.001; ****p < 0.0001. The variability is reported as standard deviation.

### Phosphatase activity of immobilized chimeras

The activity and the stability of the immobilized chimeric proteins were evaluated by measuring phosphatase activity in comparison to the immobilized native *Tt*ArsC. The specific activities were calculated on the amount of enzyme adhered on polystyrene. Interestingly, the specific activity of immobilized chimeras (ArsC-Vmh2 1.8 ± 0.1 mU/mg; Vmh2-ArsC 2.47 ± 0.03 mU/mg) resulted to be almost 5 times higher than the immobilized *Tt*ArsC (0.44 ± 0.04 mU/mg) suggesting that Vmh2 domain is critical for the adhesion (Fig. 2c; Table S1). Furthermore, the higher specific activity of Vmh2-ArsC with respect to that of ArsC-Vmh2 suggests that the catalytic moiety in Vmh2-ArsC is better exposed upon immobilization; this observation becomes more consistent also considering that when the chimeras are not immobilized, their specific activities are comparable (see above).

**Figure 3:**
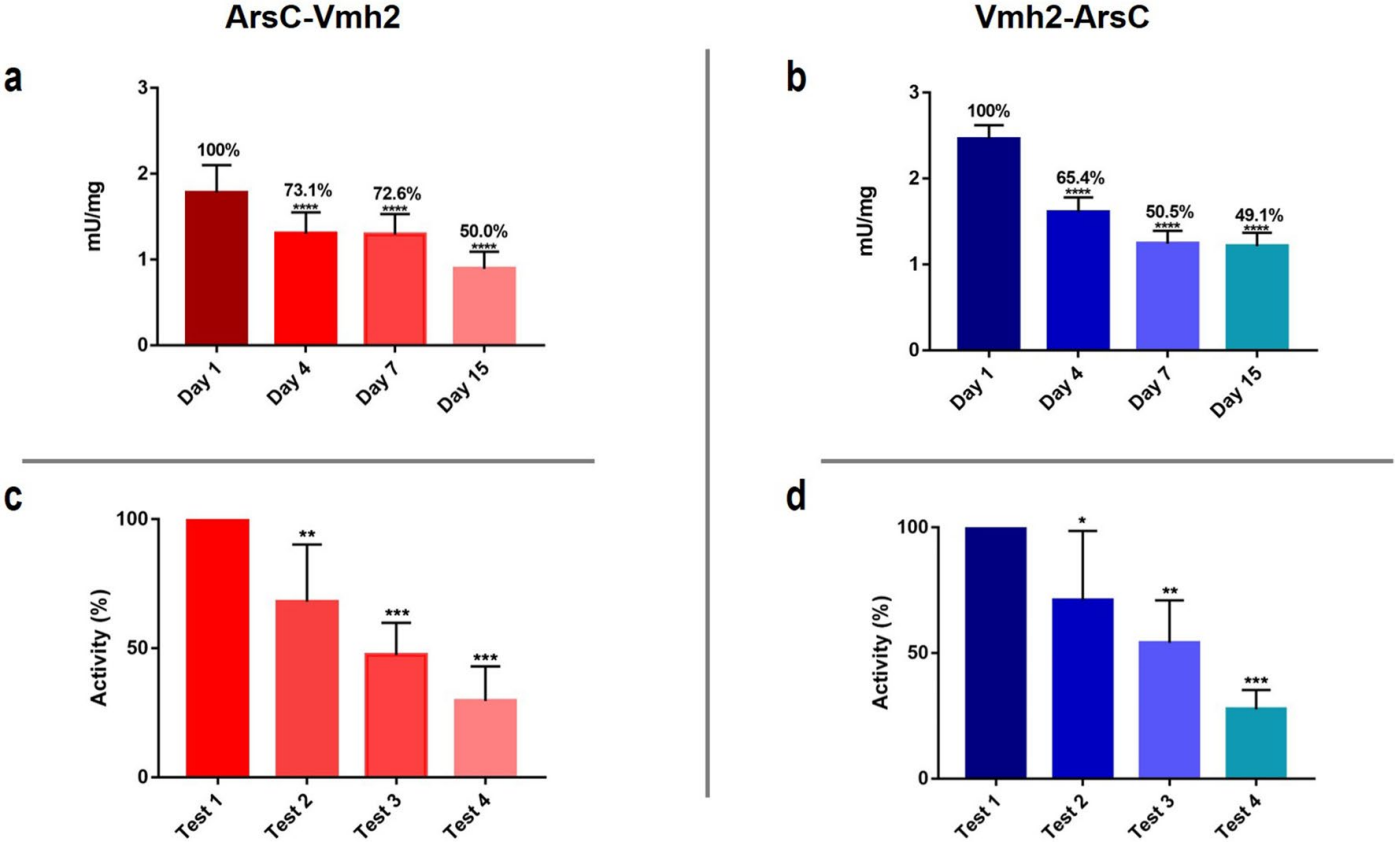
Stability evaluation of immobilized ArsC-Vmh2 (red) and Vmh2-ArsC (blue). (**a**,**b**) Functional stability of ArsC-Vmh2 and Vmh2-ArsC stored at 4 °C up to 15 days. (**c**,**d**) Residual activity of ArsC-Vmh2 and Vmh2-ArsC after *n* assays on the same immobilized proteins. Statistical analysis was performed through the ordinary one-way ANOVA on GraphPad Prism 7.00; significant differences are indicated as: *p < 0.05; **p < 0.01; ***p < 0.001; ****p < 0.0001.

The functional stability of the immobilized chimeric proteins was investigated both in terms of their aging and reuse. In particular, chimeras were adsorbed on polystyrene plates and stored for 1, 4, 7 and 15 days at 4 °C; then the residual phosphatase activity was measured. Figure 3 shows that ArsC-Vmh2 retains more activity than Vmh2-ArsC in the early days, but after 15 days both chimeras still keep about 50% of their own activity. Therefore, although Vmh2-ArsC exhibits a higher specific activity, ArsC-Vmh2 results to be more stable in the first week of storage. Furthermore, the phosphatase activity assay was repeated 4 times on the same immobilized chimeras at 1-day interval; the results indicate that both chimeras can be employed up to the third assay, with ArsC-Vmh2 keeping 47% of its specific activity and Vmh2-ArsC 54%.

Altogether these results indicate that either ArsC-Vmh2 and Vmh2-ArsC can be efficiently immobilized and maintain their catalytic function for several days.

### Immobilization on gold

**Figure 4:**
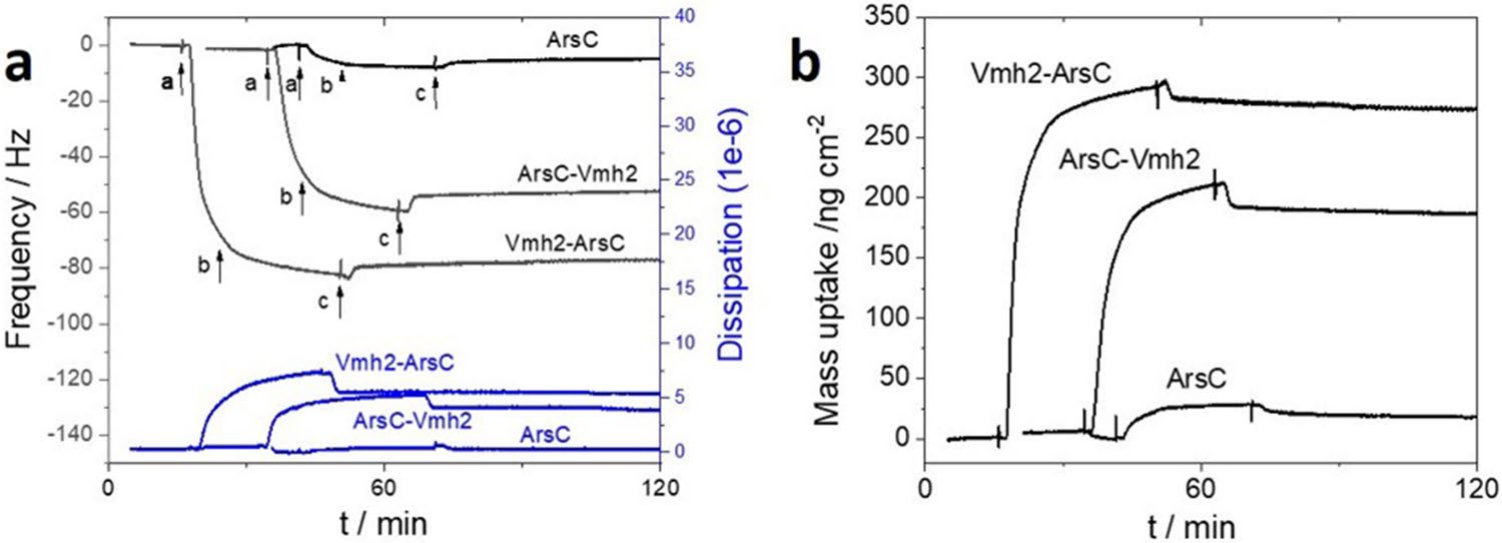
Immobilization on gold. (**a**) QCM-D profile (shifts in resonant frequency (black curve) and in dissipation (blue curve) vs. time) for the immobilization of ArsC-Vmh2, Vmh2-ArsC and ArsC (5th overtone data) before and (**a**) after injection of 0.04 mg mL^−1^ enzyme solution in Tris–HCl 50 mM, Gu-HCl 0.15 M, DTT 1 mM buffer pH 7.5 for 10 min, (**b**) stop of the flow for 20 min and (**c**) restart of the flow of Tris–HCl buffer; (**b**) Plot of enzyme mass uptake versus time calculated from the Sauerbray Eq. (1) for the 5th overtone.

Quartz Crystal Microbalance with Dissipation monitoring (QCM-D) experiments were performed in order to investigate the immobilization of ArsC-Vmh2, Vmh2-ArsC and the native *Tt*ArsC enzymes on gold-coated piezoelectric quartz crystals. A typical QCM-D profile for the three enzymes is displayed in Fig. 4a. The starting baseline corresponds to the continuous flowing of the buffer solution. As soon as Vmh2-ArsC, ArsC-Vmh2, and the native *Tt*ArsC adsorption occurs on gold surfaces, a respective frequency decrease of 82, 60 and 8 Hz is observed.

The stable interaction between ArsC-Vmh2 or Vmh2-ArsC and gold results in a stable frequency signal, even when the buffer is passed through the QCM-D cell during additional 60 min. A respective 6% and 12% frequency decrease was observed for Vmh2-ArsC and ArsC-Vmh2. This confirms the excellent stability of the Vmh2-gold interaction over time, considering that a change of the nature of the buffer in the chamber might also induce a slight decrease in frequency. On the contrary, the frequency signal corresponding to the native *Tt*ArsC exhibit 38% decrease, underlining the importance of the Vmh2 domain in stabilizing the enzyme immobilization on gold.

In parallel, the increase of the dissipation factor in the case of ArsC-Vmh2 and Vmh2-ArsC reflects a decrease in the rigidity of the gold surface, which likely arises from the homogenous formation of ArsC-Vmh2 and Vmh2-ArsC enzyme layer at the surface of gold. Figure 4b displays the plot of the mass of ArsC-Vmh2 and Vmh2-ArsC adsorbed at the surface of the gold-coated quartz as a function of time. The mass uptake of ArsC-Vmh2 and Vmh2-ArsC was estimated using the Sauerbrey Eq. (1)

1 $$\Delta m= -{C\Delta {f}_{n}}/{n},$$

where C is the mass sensitivity; C = 17.7 ng cm^−2^ Hz^−1^ at f_1_ = 5 MHz and n is the accordant overtone number. Curves for each overtone show an average mass of immobilized ArsC-Vmh2 and Vmh2-ArsC of 187 ± 22 and 275 ± 5 ng cm^−2^ respectively. Considering a 50% degree of hydration for the enzyme layer^59^, this would correspond to a ArsC-Vmh2 and Vmh2-ArsC surface density of 4.3 and 6.4 pmol cm^−2^ respectively. As expected, a low amount of native *Tt*ArsC (0.7 pmol cm^−2^) was adsorbed on the surface of gold. In addition to high amount of immobilized enzymes, it is also noteworthy that, according to the initial mass uptake evolution, an initial rate of deposition of 2.4 and 1.0 pmol cm^−2^ min^−1^ was measured for Vmh2-ArsC and ArsC-Vmh2 respectively. This rate is almost ten-times higher compared to the simple adsorption of the native enzyme (0.25 pmol cm^−2^ min^−1^), hypothesizing that *Tt*ArsC, Vmh2-ArsC and ArsC-Vmh2 might have the same flow rate into the cell.

### Electrochemical As(III) biosensor

**Figure 5:**
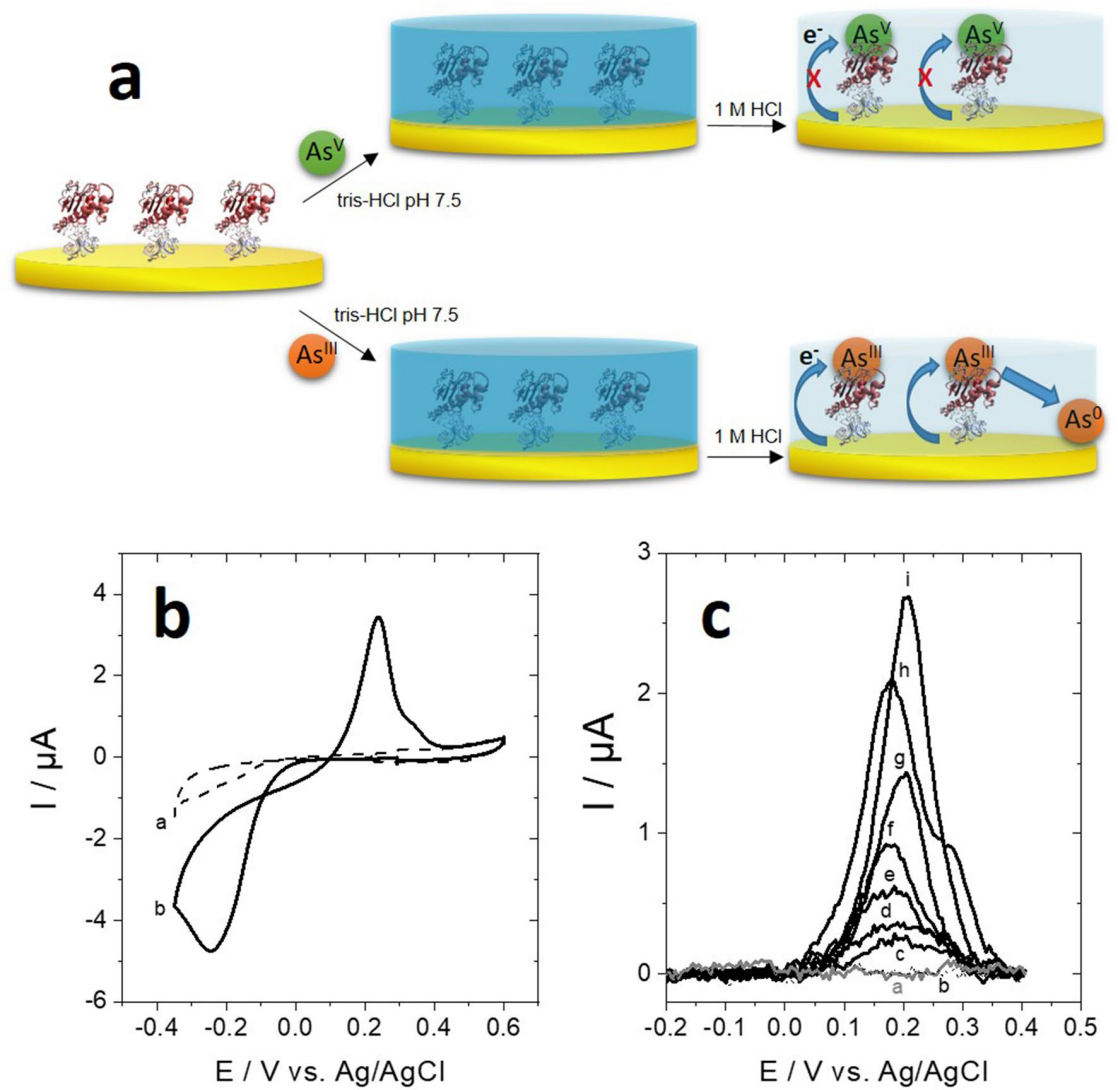
Electrochemical arsenic biosensor. (**a**) Schematic representation of the binding of As(III) and As(V) at Vmh2-ArsC modified gold electrodes. Down: (**b**) CV of the (**a**) nonmodified gold electrode and (**b**) Vmh2-ArsC-modified gold electrodes after incubation (1 h, 60 °C) in solutions of 0.5 mM As(III) and transfer in 1 M HCl, range (− 0.4 to + 0.6) V versus Ag/AgCl, scan rate 10 mV s^−1^. (**c**) SWV of the Vmh2-ArsC-modified gold electrodes after incubation (1 h, 60 °C) in solutions of 0.5 mM As(III) for the (**a**, gray) nonmodified gold electrode, (**b**, dashed line) Vmh2-modified gold electrode and (**c**) 0.1, (**d**) 0.25, (**e**) 0.5, (**f**) 0.75, (**g**) 1, (**h**) 5 and (**i**) 10 mM As(III) for the Vmh2-ArsC-modified gold electrode. SWV parameter: 1 M HCl, pre-deposition at − 0.4 V versus SCE for 5 min, scan rate 100 mV s^−1^, f = 50 Hz.

Vmh2-ArsC and ArsC-Vmh2-modified gold electrodes were then investigated towards arsenate and arsenite capture and electrochemical detection by anodic stripping voltammetry. *Tt*ArsC has already proven its ability to interact with both arsenate and arsenite^24,25^. Here, owing to the ability of Vmh2-ArsC and ArsC-Vmh2 to strongly interact with gold surfaces, we modified gold electrodes with both Vmh2-ArsC and ArsC-Vmh2. Binding of both arsenate and arsenite were investigated at these enzyme electrodes (Fig. 5a). The biofunctionalized gold was used in a first step to attach arsenic at pH 7.5. Then, the electrode was transferred in a 1 M HCl solution for anodic stripping voltammetry and arsenic detection by Square-Wave voltammetry (SWV). This procedure differs from most of arsenic electrochemical sensors by the fact that ArsC-modified gold electrode is used to attach As(III) to the electrode at pH 7.5, before its detection in 1 M HCl, thus providing an alternative strategy to separate As(III) from the sample before its detection by SWV. In the majority of ASV-based methods As(0) is directly electrodeposited from As(III) sample solution, potentially inducing unwanted stripping peaks and intermetallic deposits^60^. Since gold has no affinity for As(III), either the unmodified gold electrode and the one modified with Vmh2 alone, do not exhibit signals corresponding to arsenic reduction after transfer of the arsenic solution to the 1 M HCl solution (Fig. 5b,c). After soaking the electrode in a 10 mM solution of potassium arsenate and transfer in the 1 M HCl solution, no detection of arsenate reduction into arsenite was observed at gold electrodes. This is mostly due to the fact that As(V) cannot be electrochemically-reduced into As(III) at electrodes^60,61^. The modified gold electrode was also soaked in a 10 mM solution of sodium (meta)arsenite (NaAsO_2_). Figure 5b displays the CV of a Vmh2-ArsC-modified electrode in 1 M HCl.

**Figure 6:**
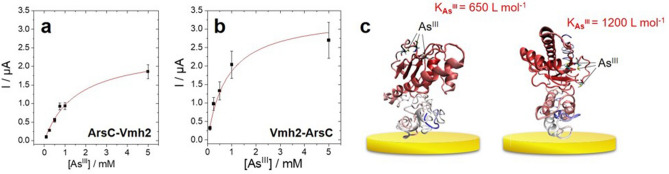
Evolution of the SWV peak current towards starting As(III) concentrations. (**a**) ArsC-Vmh2-modified gold electrode and (**b**) Vmh2-ArsC-modified gold electrodes accompanied by a schematic representation of arsenic binding (**c**). The red line corresponds to the curve fitting using Eq. (2).

The shape of the CV is indicative of the presence of As(III) at the surface of the electrode (Fig. 5b). This shape is typically observed in anodic stripping voltammetry experiments performed on gold electrodes in acidic As(III) solutions^62–64^. An irreversible reduction wave at − 0.25 V versus Ag/AgCl corresponds to the reduction of As(III) into As(0) which is subsequently oxidized on the reversed scan at + 0.24 V. It is clear that As(III) is immobilized owing to the presence of Vmh2-ArsC and ArsC-Vmh2 at the surface of the electrode. No As(III) was detected on a non-modified gold electrode after soaking the electrode in an As(III) solution. Then, to characterize the interaction between bioelectrodes and arsenite and to examine the ability of the bioelectrode to detect different concentrations of As(III) in water, electrochemical detection was performed at different concentrations using SWV. Figure 5c displays SWV performed after the incubation step (electrodes placed inside solutions with increasing concentrations of arsenic) and the reduction step which fully reduces the attached As(III) into As(0). A shoulder peak might be observed during SWV experiments as observed at 5 mM As(III). This likely arises from the inhomogeneity of As(III) binding on the enzyme layer at higher concentrations. Figure 6 shows the evolution of the peak current towards increasing concentration of arsenic.

Increasing peak current follows a classic Langmuir isotherm model for both immobilized enzymes, which was confidently modelled according to Eq. (2)

2 $${I}_{p,eq}= \frac{{I}_{p,max} \times {K}_{{{\varvec{A}}{\varvec{s}}}^{{\varvec{I}}{\varvec{I}}{\varvec{I}}}}\times [{{\varvec{A}}{\varvec{s}}}^{{\varvec{I}}{\varvec{I}}{\varvec{I}}}]}{1+{K}_{{{\varvec{A}}{\varvec{s}}}^{{\varvec{I}}{\varvec{I}}{\varvec{I}}}}\times [{{\varvec{A}}{\varvec{s}}}^{{\varvec{I}}{\varvec{I}}{\varvec{I}}}]}$$

where I_p,eq_ is the equilibrium peak current, I_p,max_ is the peak current at saturating concentrations of As^III^ and K_**As**_^**III**^ is the association constant between As^**III**^ and the immobilized enzyme. For ArsC-Vmh2, the best fit was achieved with a I_p,max_ = 2.5 (± 0.2) μA and K_**As**_^**III**^ = 650 (± 100) L mol^−1^ at 60 °C in 50 mM Tris–HCl pH 7.5 buffer. For Vmh2-ArsC, the best fit was achieved with a I_p,max_ = 3.4 (± 0.2) μA and K_**As**_^**III**^ = 1200 (± 300) L mol^−1^ . Higher I_p.max_ of Vmh2-ArsC indicates that more enzyme is immobilized. This is in good agreement with QCM-D experiments, indicating that Vmh2-ArsC has higher affinity for the surface of gold as compared to ArsC-Vmh2. This might arise from a more accessible Vmh2 domain in the folded Vmh2-ArsC chimera able to interact with surfaces. In addition, higher affinity constant for Vmh2-ArsC might indicate that the *Tt*ArsC domain is more accessible to arsenic binding as compared to immobilized ArsC-Vmh2, confirming the hypothesis that once immobilized Vmh2-ArsC exposes the catalytic moiety better than ArsC-Vmh2. The highly acidic condition of As(III) detection is detrimental to the proteins structure and therefore this kind of biosensor can be used as a single-use sensor. However, owing to the efficient immobilization process and the low amount of the fusion proteins required, the gold electrode surface can be easily re-functionalized.

## Conclusions

In this work we developed an electrochemical biosensor based on chimeric proteins endowed with both the adhesive properties of Vmh2, a self-assembling amyloid protein^39^ and the arsenic sensing ability of a thermostable arsenate reductase, *Tt*ArsC^13^. Two chimeric genes coding for two alternative fusion proteins (ArsC-Vmh2 and Vmh2-ArsC) were designed, heterologously produced and characterised for their activity and their ability to be immobilized on polystyrene and gold. The results suggest that the position of Vmh2 at the N-terminal of the chimera has a positive effect on its folding, its yield of production (higher for Vmh2-ArsC) and renaturation (higher for Vmh2-ArsC). Furthermore, we observed that the catalytic activity of Vmh2-ArsC is slightly higher than that of ArsC-Vmh2 only when the proteins are immobilized. This result suggests that the expression of Vmh2 at the N-terminal of a chimeric protein^42–44^ favors the anchorage on a surface, which improves catalytic activity in terms of substrate accessibility. This is the first time that Vmh2 is expressed at the N-terminal of a chimeric protein^42–44^. Both chimeras demonstrated to be very stable since, once immobilized, they retained their catalytic function for several days and could be reused up to three times; their high stability could be due to the thermophilic origin of *Tt*ArsC and its intrinsic higher resistance to harsh conditions.

Moreover, the chimeras were used to modify the surface of gold electrodes in order to build an As(III)-sensitive bioelectrode. The results confirm the difference on the substrate accessibility of the immobilized chimeras, which is higher for Vmh2-ArsC. Owing to the interaction of immobilized ArsC and As(III), As(III) was successfully extracted from neutral pH solutions and electroreduced at acidic pHs. Despite the fact that maximum current densities, as well as binding constants have to be increased in order to possibly use these bioelectrodes for As(III) biosensing applications^3,31^, this work represents the first example of the use of a thermostable arsenate reductase in an enzyme-based electrochemical arsenic biosensor. The novel use of chimeric enzymes able to provide either a recognition step for As(III) followed by its detection by SWV and an increased ability to bind to hydrophobic surfaces represents an original and promising alternative for arsenic sensing, paving the way to the use of ArsC-Vmh2 and Vmh2-ArsC as a new platform for biosensing in environmental applications. These improvements can be achieved either by further enzyme engineering strategies or by the use of advanced nanostructured electrodes.

## Methods

### Gene synthesis

Two different gene fusions were designed in order to obtain two versions of the chimeric protein based on the arsenate reductase *Tt*ArsC and the hydrophobin Vmh2; the first (*arsC-vmh2*) presents the gene coding for *Tt*ArsC at 5′end, and the gene coding for Vmh2 at the 3′end; on the other hand, the second (*vmh2-arsC*) presents *vmh2* at the 5′end and *arsC* at the 3′end. Both the synthetic genes were designed to possess a sequence coding for a flexible linker^65^ of 15 amino acids between the two proteins and the sequence coding for the thrombin cleavage site (LVPRGS) at the 3′end. The synthetic genes were designed with the GeneArt tool, optimized according to the *E. coli* codon usage and ordered at Thermo Fisher Scientific (https://www.thermofisher.com/it/en/home/life-science/cloning/gene-synthesis/geneart-gene-synthesis.html).

### Bioinformatic analysis

Theoretical isoelectric points and molecular weights of chimeric proteins were determined using the ProtParam tool of ExPASy (https://web.expasy.org/protparam/). Three-dimensional structure models were elaborated using I-TASSER^66^ (https://zhanglab.ccmb.med.umich.edu/I-TASSER/) and the quality of the predicted models was estimated by determining the confidence score (C-score). It is calculated based on the significance of threading template alignments and the convergence parameters of the structure assembly simulations. C-score is typically in the range of [− 5, 2], where a C-score of a higher value indicates a model with high confidence^46,47^. The 3D-models were visualized using PyMOL v0.99 (https://pymol.org/2/). The propensity to aggregation of chimeras was evaluated on TANGO (http://tango.crg.es/), a computer algorithm for prediction of aggregating regions in unfolded polypeptide chains.

### Cloning and heterologous expression

The synthetic genes were cloned in the pET28b(+) expression vectors (Novagen), between *NcoI* and *HindIII* restriction sites, to insert the His-tag at the C-terminal of the resulting proteins. Colonies of *E. coli* Top10F’ transformed with pET28b(+)/*arsC-vmh2* and pET28b(+)/*vmh2-arsC* (Supplementary Fig. S3) were screened by PCR colony using the primer pairs chArsC FW, chVmh2 RV for *arsC-vmh2* and chVmh2 FW, chArsC RV for *vmh2-arsC* and Taq DNA Pol (Thermo Scientific) (Table 2). To verify the appropriate insertions, plasmids were recovered from the recombinant colonies using the QIAprep spin Miniprep Kit (QIAGEN) and digested with Nco*I*-HF and Hind*III*-HF (New England Biolabs).

**Table 2:** Primers employed for PCR colony.

**arsC-vmh2**	
chArsC FW	5′ ATGCGTGTTCTGGTTCTGTG 3′
chVmh2 RV	5′ CAGGCTAATGTTAATCGGGCTG 3′
**vmh2-arsC**	
chVmh2 FW	5′ ATGGACACCCCGAGCTGTAGCACC 3′
chArsC RV	5′ CAGGCGTGCTGCCTGACGCA 3′

For the protein expression, *E. coli* BL21 (DE3) was transformed with the recombinant vectors. Colonies were grown for 16 h in LB medium containing kanamycin (50 μg/L), and chloramphenicol (33 μg mL/L) then diluted in 1 L of fresh medium with antibiotics. When the culture reached 0.6 OD_600 nm_, protein expression was induced by the addition of 1 mM isopropyl-1-thio-β-d-galactopyranoside (IPTG) and the bacterial cultures were grown for additional 6 h at 37 °C.

### Purification of chimeras

#### Denaturation of inclusion bodies

Cells were harvested by centrifugation and pellets were resuspended in lysis buffer (Tris–HCl 50 mM pH 8; EDTA 10 mM) and sonicated for 15 min; the sonicator (MISONIX MOD.XL2020) was set up at 35% of amplitude with pulses of 30″ on and 30″ off. The lysate was centrifuged at 15,000 rpm for 60 min (JA25.50 rotor; Beckman). Inclusion bodies were resuspended in Tris–HCl 100 mM pH 8, EDTA 10 mM, Triton X-100 2%, urea 2 M, sonicated for 2 min and centrifugated for 30 min at 15,000 rpm (JA25.50 rotor; Beckman); this step was repeated twice. After 3 washings in Tris–HCl 100 mM pH 8 (preceded each time by 2 min sonication and centrifugation for 30 min), inclusion bodies were resuspended in denaturation buffer (Tris–HCl 50 mM pH 8, Gu-HCl 6 M) and sonicated for further 15 min. Inclusion bodies were then kept under shaking at 37 °C for 16 h after adding β-mercaptoethanol 20 mM.

#### Renaturation of chimeras

DTT 2 mM was added to the solubilized inclusion bodies, and incubation prolonged at room temperature for 2 h. The chimeric proteins were refolded by diluting tenfold the solution containing the denatured inclusion bodies solution through addition of renaturation buffer drop by drop. In order to find the best conditions for protein renaturation different refolding buffers were used and they are reported in Table 3. The solutions were then centrifuged for 1 h at 15,000 rpm (JA25.50 rotor; Beckman), and the supernatants were concentrated using an Amicon ultrafiltration system with 3 kDa filters (ULTRACEL Millipore) to get a final concentration of Gu-HCl of 0.15 M and finally dialyzed alternatively against buffer A_D_, B_D_, or C_D_ (Table 3).

**Table 3:** List of conditions used to refold ArsC-Vmh2 and Vmh2-ArsC.

Conditions	Refolding buffers		Dialysis buffers	
A	A_R_	Tris–HCl 100 mM pH 7.5, EDTA 10 mM, NaCl 0.3 M, l-Arginine 0.5 M, DTT 1 mM	A_D_	Tris–HCl 50 mM pH 7.5, DTT 1 mM
B	B_R_	Sodium-Phosphate 100 mM pH 7.5, EDTA 10 mM, NaCl 0.3 M, l-Arginine 0.5 M, DTT 1 mM	B_D_	Sodium-Phosphate 50 mM pH 7.5, DTT 1 mM
C	C_R_	Tris–HCl 100 mM pH 7.5, EDTA 10 mM, NaCl 0.3 M, l-Arginine 0.5 M, DTT 1 mM	C_D_	Tris–HCl 50 mM pH 7.5, Et-OH 40%, DTT 1 mM

### Analytical methods

Protein concentration of denatured inclusion bodies and of purified ArsC-Vmh2 and Vmh2-ArsC was determined using the Pierce 660 method (Thermo Fischer Scientific, Waltham, Massachusetts, USA) according to the manufacturer’s instructions and using bovine serum albumin (BSA) as the standard.

Protein purification was estimated by SDS-PAGE performed on 12% (w/v) gels supplemented with urea 36%^67^; protein identification was determined by Western blotting performed using (4 μg) of proteins and following standard procedures^68,69^. The detection was performed using monoclonal anti-polyHistidine antibody (1:10,000) (Sigma-Aldrich), and chemiluminescent reaction with the “Immobilon Western chemiluminescent horseradish peroxidase (HRP)” kit (Millipore). Acquisitions were developed with ChemiDoc XRS (Bio-Rad).

### Immobilization on polystyrene plate

100 μL of ArsC-Vmh2 and Vmh2-ArsC at different concentrations (0.025; 0.05; 0.1; 0.2 mg/mL) were deposited into the wells of polystyrene multi-well plates (96 wells) and incubated at 28 °C for 16 h. Controls were obtained by depositing 100 μL of renaturation buffer A (50 mM Tris–HCl pH 7.5, 0.15 M GuHCl, 1 mM DTT) or 100 μL of purified *Tt*ArsC at the above indicated concentrations. After incubation, to eliminate unbound proteins, the wells were washed three times with 100 μL of 50 mM Tris–HCl, pH 7.5. The amount of immobilized proteins was calculated by subtracting the amount of unbound proteins (determined through the Pierce 660 method) and the immobilization yield was calculated as the ratio (%) between μg of immobilized proteins and μg of proteins initially deposited into the wells. All the experiments were performed in triplicates. Statistical analysis was performed through the ordinary one-way ANOVA on GraphPad Prism 7.00; significant differences of TtArsC in comparison to chimeras are indicated as: *p < 0.05; **p < 0.01; ***p < 0.001; ****p < 0.0001.

$$\begin{aligned} &Immobilized\,proteins=Deposited\,proteins-Unbound\,proteins\\ &Yield\,of\,immobilization=\frac{Immobilized\,proteins}{Deposited\,proteins}\% \end{aligned}$$

### Arsenate reductase activity assay

Arsenate reductase activity was measured using a coupled assay that follows the arsenate dependent oxidation of NADPH in the presence of Trx from *E. coli* and Tr from *Sulfolobus solfataricus* (*Ss*Tr) both purified following reported procedures^55,56^. A typical assay was performed in a final reaction volume of 160 μL at 60 °C in a double beam spectrophotometer (Cary 100, Varian). The arsenate reduction activity was measured by following the decrease in absorption at 340 nm due to the oxidation of NADPH^12^ as described by Del Giudice et al.^13^. One unit of enzyme activity (U) was defined as the amount of enzyme required to consume 1 μmol NADPH per minute, under the assay condition described. The specific activity is reported as units of enzyme activity per milligram of enzyme (U/mg). Each reaction was performed in triplicate, and in the same buffer condition.

### Phosphatase activity assay

The phosphatase activity of chimeras and of chimeras immobilized in polystyrene plates was measured at 60 °C using pNPP as substrate (Sigma-Aldrich) and following the increase in absorption at 405 nm due to the formation of *p*-nitrophenol (Δε405 = 18,000 M^−1^ cm^−1^)^13^. Each reaction was performed in triplicate in a plate reader spectrophotometer (Sinergy H4, software version 2.07.17) in a total volume of 160 μL containing 4 μM of ArsC-Vmh2 or Vmh2-ArsC, 60 mM pNPP in 50 mM Tris–HCl pH 7.0. As controls the same reactions were performed: (a) in the absence of enzyme, (b) with immobilized *Tt*ArsC (4 μM). The variation of Abs_405nm_ per minute (∆OD/min) obtained in the absence of enzyme was subtracted to that one obtained from the experiments with enzymes. One unit of enzyme activity (U) was defined as the amount of enzyme required to release 1 μmol *p*-nitrophenol *per* minute, under the assay condition described. The specific activity of immobilized proteins was calculated as enzyme units per milligram of proteins adhered to polystyrene as determined above (Immobilization on polystyrene plate).

### Quartz-crystal microbalance analysis with dissipation monitoring (QCM-D)

QCM-D measurements were performed using E1 instruments (Q-Sense, AB, Göteborg, Sweden) equipped with one laminar flow chambers and polished AT-cut piezoelectric quartz crystals (diam. 14 mm) covered by a 100 nm thick gold layer (QSX 301-Q-Sense). f and D were measured at the fundamental resonance frequency (5 MHz) as well as at the third, fifth, seventh, ninth, eleventh, and thirteenth overtones (n = 3, 5, 7, 9, 11 and 13). Experiments were conducted in a continuous flow at a flow rate of 50 μL min^−1^. For each experiment the instrument was equilibrated with 50 mM Tris–HCl pH 7.5, 0.15 M Gu-HCl, 1 mM DTT buffer. After 15 min of signal stabilization, 500 μL of the enzyme solution (0.04 mg mL^−1^ in buffer 50 mM Tris–HCl pH 7.5, 0.15 M Gu-HCl, 1 mM DTT) was injected during 10 min (50 μL/min) and then the flow stopped for 20 min before flowing the buffer solution to remove the unbound enzyme, until signal stabilization. Each set of experiment was performed in duplicate.

### Electrochemical analysis

The electrochemical experiments were carried out in a three electrodes electrochemical cell using a Biologic VMP3 Multi Potentiostat. Gold electrodes (0.071 cm^2^) were used as working electrodes. Pt wire was used as counter electrode and a silver chloride electrode (Ag/AgCl) served as reference electrode. All potentials are given versus Ag/AgCl.

To immobilize ArsC-Vmh2 and Vmh2-ArsC on gold electrodes 20 μL of chimeric enzymes (0.2 mg mL^−1^) were deposited on the electrode surfaces, and dried at 4 °C overnight. As negative controls, *Tt*ArsC and Vmh2 were used. The electrodes were then washed with 50 mM Tris–HCl pH 7.5 and incubated for 1 h at 60 °C in different solutions of sodium (meta)arsenite (NaAsO_2_) ranging from 0.1 to 5 mM. After incubation, Cyclic voltammetry (CV) and Square Wave Voltammetry (SWV) experiments were performed in 1 M HCl solution. Each set of experiment was performed in triplicate.

## Supplementary Information

Supplementary Informations.
