# Supplementary material for: Self-assembling thermostable chimeras as new platform for arsenic biosensing
Source: Sci Rep. 2021 Feb 4;11:2991. doi: 10.1038/s41598-021-82648-9 (PMC7862302; doi:10.1038/s41598-021-82648-9)
Supplement: Supplementary file 1 — Supplementary Informations. [file 41598_2021_82648_MOESM1_ESM.pdf]

Self-assembling thermostable chimeras as new platform for arsenic biosensing

Rosanna Puopolo[1], Ilaria Sorrentino[2], Giovanni Gallo[1], Alessandra Piscitelli[3], Paola Giardina[3], Alan Le Goff[2]*, Gabriella Fiorentino[1]*

*corresponding authors

alan.le-goff@univ-grenoble-alpes.fr

fiogabri@unina.it

[1] Department of Biology, University of Naples Federico II, 80126 Naples, Italy;

[2] Department of Molecular Chemistry, University Grenoble Alpes, CNRS, 38000 Grenoble, France;

[3] Department of Chemical Sciences, University of Naples Federico II, 80126 Naples, Italy

Supplementary figures

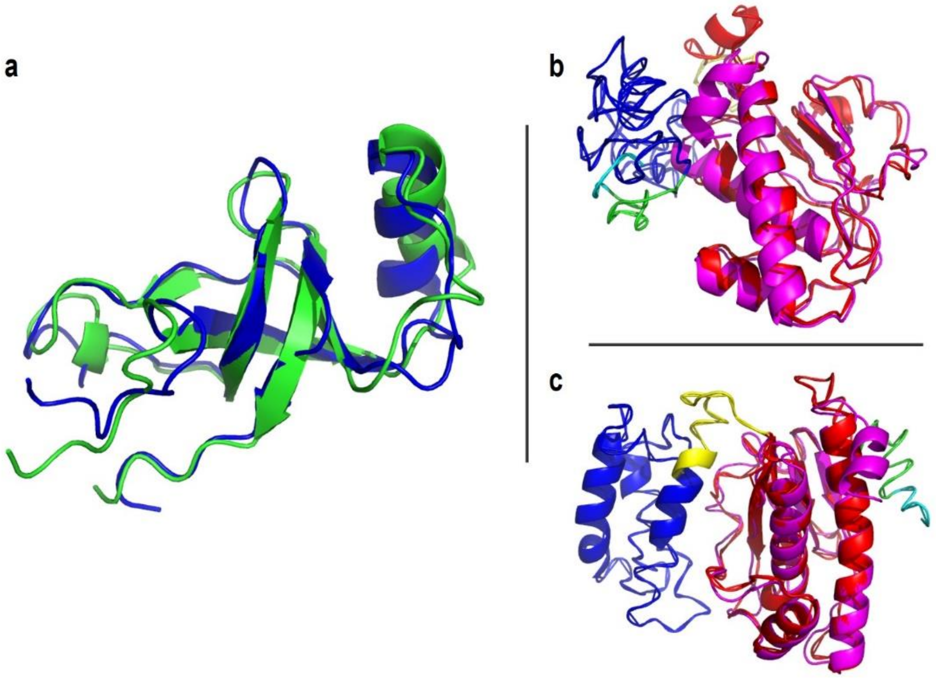

Figure S1. **Comparison of 3D models.** (**a**) Overlap of the 3D models of Vmh2: in green the model published by Pennacchio *et al*.(2018); in blue the model elaborated on I-TASSER in this work; Overlap of the 3D model of *Tt*ArsC (magenta) on ArsC-Vmh2 (**b**) and on Vmh2-ArsC (**c**). Chimeras code colour: ArsC (red), Vmh2 (blue), linker (yellow), Thrombin cleavage site (green), His-tag (ciano).

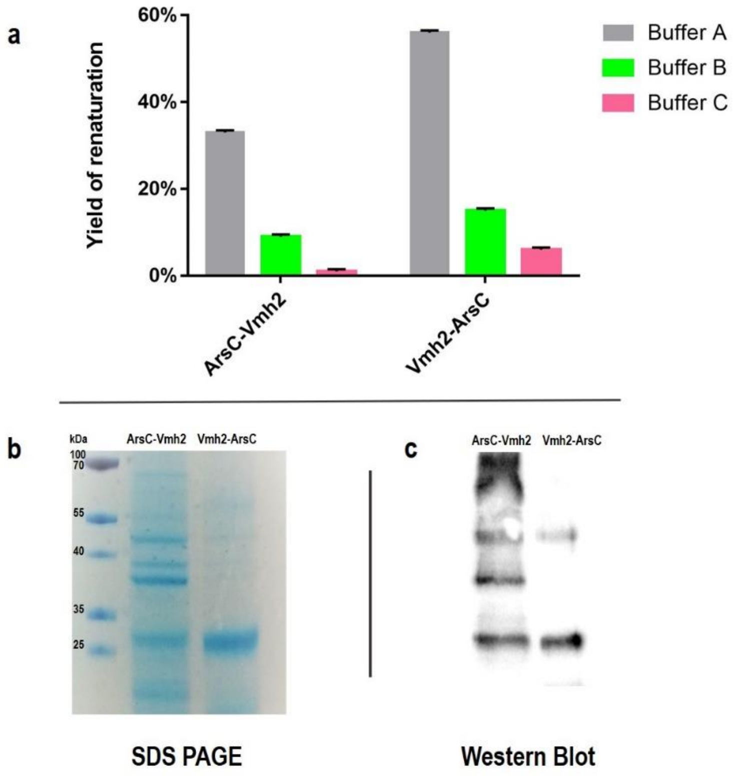

Figure S2. ArsC-Vmh2 and Vmh2-ArsC purification analysis. (**a**) Yield of renaturation of chimeric proteins from inclusion bodies in different buffers after dialysis. Buffer A = Tris-HCl 50mM pH 7.5, Gu-HCl 0.15 M; Buffer B = Sodium Phosphate 50mM pH 7.5, Gu-HCl 0.15 M; Buffer C = Tris-HCl 50 mM pH 7.5, Et-OH 40%, Gu-HCl 0.15 M. (**b**) SDS PAGE of ArsC-Vmh2 (4 µg) and Vmh2-ArsC (4 µg). (**c**) Western blot of ArsC-Vmh2 (4 µg) and Vmh2-ArsC (4 µg), with anti-polyHistidine antibody. Full length gel and blot are shown in Supplementary Fig. S4.

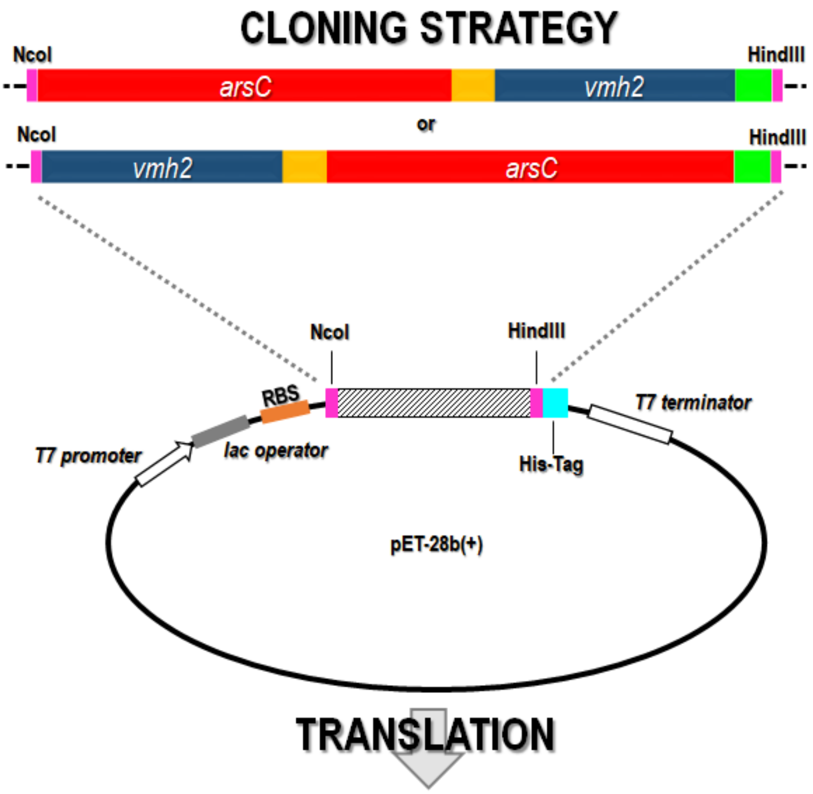

MGMRVLVLCTHNSARSQMAEAWLKHFARELGVDLEVHSAGTEKAFVKEEAKRVMAEVGLDLSGHFSKTLFEVPDPWDFGLVLTVCDQAKEACPAYPAR
TEKRHVSFPDPTGKPLEEWRKVRDALGRMARFLVEALKEGRLPSDEELRQAARLGGGGSGGGGSGGGGSMDTPSCSTGSLQCCSSVQKATDPLASLLI
GLLGIVLGPLDLLVGVTCSPITVIGVGGTSCTQQTVCCTGNSFNGLIAIGCSPINISLLVPRGSKLAAALEHHHHHH

or

MDTPSCSTGSLQCCSSVQKATDPLASLLIGLLGIVLGPLDLLVGVTCSPITVIGVGGTSCTQQTVCCTGNSFNGLIAIGCSPINISLGGGGSGGGGSGGGGS
MRVLVLCTHNSARSQMAEAWLKHFARELGVDLEVHSAGTEKAFVKEEAKRVMAEVGLDLSGHFSKTLFEVPDPWDFGLVLTVCDQAKEACPAYPARTEK
RHVSFPDPTGKPLEEWRKVRDALGRMARFLVEALKEGRLPSDEELRQAARLLVPRGSKLAAALEHHHHHH

Figure S3. Representation of the cloning strategy for the expression of ArsC-Vmh2 and Vmh2-ArsC. The synthetic genes coding for ArsC-Vmh2 and Vmh2-ArsC were cloned in the expression vector pET-28b(+) between the NcoI and HindIII sites, giving the plasmids named *pET-28b(+)/arsC-vmh2* and *pET-28b(+)/vmh2-arsC*; they were used to transform *E. coli* BL21 (DE3).

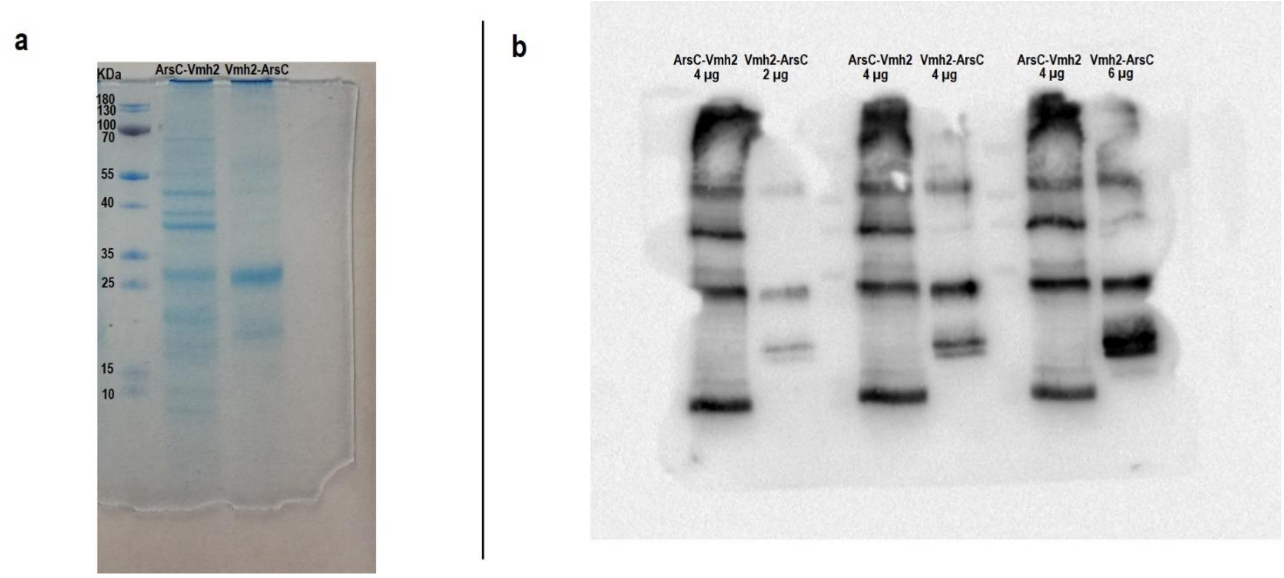

Figure S4. Western blot analysis a) SDS PAGE of ArsC-Vmh2 (4 µg) and Vmh2-ArsC (4 µg). (**b**) Western blot of ArsC-Vmh2 and Vmh2-ArsC with anti-polyHistidine antibody. This figure is the full-length version of the cropped SDS PAGE and immunoblot in Supplementary Fig. S2.

Supplementary table

Table S1. The specific activities of *Tt*ArsC, ArsC-Vmh2 and Vmh2-ArsC converted from mU/mg to mU/nmol. Standard deviation is reported for each data set.

	As(V) reductase activity				Phosphatase activity (in solution)				Phosphatase activity (immobilized)			
	$\frac{mU}{mg}$	±	$\frac{mU}{nmol}$	±	$\frac{mU}{mg}$	±	$\frac{mU}{nmol}$	±	$\frac{mU}{mg}$	±	$\frac{mU}{nmol}$	±
***Tt*ArsC**	250	10	4.3	0.2	15	2	0.26	0.03	0.44	0.04	0.007	0.001
**ArsC-Vmh2**	200	10	5.8	0.3	2.7	0.5	0.08	0.01	1.8	0.1	0.052	0.003
**Vmh2-ArsC**	170	10	4.9	0.3	2.2	0.5	0.06	0.01	2.47	0.03	0.071	0.001
